# Receptor-mediated Uptake of Folic Acid-functionalized Dextran Nanoparticles for Applications in Photodynamic Therapy

**DOI:** 10.3390/polym11050896

**Published:** 2019-05-16

**Authors:** Kathrin Butzbach, Matthias Konhäuser, Matthias Fach, Denise N. Bamberger, Benjamin Breitenbach, Bernd Epe, Peter R. Wich

**Affiliations:** 1 Institute of Pharmacy and Biochemistry, Johannes Gutenberg University, Staudingerweg 5, 55128 Mainz, Germany; 2 Department of Health Technology, Technical University of Denmark, Produktionstorvet Building 423, 2800 Lyngby, Denmark; 3 School of Chemical Engineering, University of New South Wales, Science and Engineering Building, Sydney, NSW 2052, Australia; 4 Australian Centre for NanoMedicine, University of New South Wales, Sydney, NSW 2052, Australia

**Keywords:** nanoparticles, dextran, targeting, folic acid, photodynamic therapy

## Abstract

In photodynamic therapy (PDT), photosensitizers and light are used to cause photochemically induced cell death. The selectivity and the effectiveness of the phototoxicity in cancer can be increased by a specific uptake of the photosensitizer into tumor cells. A promising target for this goal is the folic acid receptor α (FRα), which is overexpressed on the surface of many tumor cells and mediates an endocytotic uptake. Here, we describe a polysaccharide-based nanoparticle system suitable for targeted uptake and its photochemical and photobiological characterization. The photosensitizer 5, 10, 15, 20-tetraphenyl-21H, 23H-porphyrine (TPP) was encapsulated in spermine- and acetal-modified dextran (SpAcDex) nanoparticles and conjugated with folic acid (FA) on the surface [SpAcDex(TPP)-FA]. The particles are successfully taken up by human HeLa-KB cells, and a light-induced cytotoxicity is observable. An excess of free folate as the competitor for the FRα-mediated uptake inhibits the phototoxicity. In conclusion, folate-modified SpAcDex particles are a promising drug delivery system for a tumor cell targeted photodynamic therapy.

## 1. Introduction

Cancer remains one of the leading causes of deaths worldwide [1]. A major reason for this is the current inability to address chemotherapeutics selectively to the diseased tissue without provoking cytotoxic effects in healthy cells. Nanoparticles (NP) have the potential of overcoming this major limitation of chemotherapeutics by using them for targeted therapies in which the active substance is specifically delivered to the specified organ, tissue, or cell population, thereby increasing its therapeutic efficacy while reducing side effects [2].

Spermine and acetal modified dextran (SpAcDex) NPs represent a promising class of biocompatible particles for this task [3,4,5,6]. Dextran is a non-toxic and biocompatible safe polysaccharide biopolymer that is used in various clinical applications, for example, as a plasma expander [7]. The acetalation of dextran leads to an insolubility in water but increases the solubility in organic solvents. This solubility switch enables a particle formation using emulsion techniques in which a big hydrophilic or a small hydrophobic drug can be encapsulated [4,8,9,10]. Under mild acid conditions, for example, after cellular uptake in the late endosomes and lysosomes, hydrolysis of the acetals occurs, and the particle material reassumes its native water-soluble form and can release the entrapped payload [3]. Another advantage of the system is the cationic character of the spermine-modification, which increases the general cellular-uptake and allows additional useful modifications of the particle surface [11,12].

The system is particularly efficient for the encapsulation and the transport of hydrophobic drugs, such as the 5, 10, 15, 20-tetraphenyl-21H, 23H-porphyrine (TPP) selected as a payload for this study. It is used as a highly efficient photosensitizer in photodynamic therapy (PDT) but has a poor water solubility, which limits is effectiveness in therapeutic applications [13]. PDT makes use of the fact that photosensitizers are only cytotoxic for cells when irradiated with light [14]. Activation of TPP by light leads to the production of singlet oxygen and radical species, resulting in direct tumor cell killing, inflammatory responses, and damage to the microvasculature of the tumor [15]. Unfortunately, most hydrophobic photosensitizers accumulate after bloodstream injection not only in the tumor, but also in normal tissue [16]. This leads to harmful side effects in healthy cells near the local irradiation side. A targeted delivery of the photosensitizer could solve this problem through an enhanced phototoxicity as a result of higher and more selective accumulation in the tumor cells [17]. This can potentially be achieved by addressing the high affinity folate receptor FRα, which is able to perform receptor-mediated endocytosis and is highly overexpressed on the surface of many tumor cells [18,19]. These properties can be exploited by targeting NPs that are decorated with folic acid (FA) on the surface to result in an increased endosomal uptake [20,21]. The targeting of the nanoparticles is further increased by the so-called enhanced permeability and retention (EPR) effect that is characteristic for tumor tissue [22,23].

To test whether the FRα-mediated uptake can be exploited with nanoparticles for a tumor-targeted PDT, we covalently bound folic acid on the surface of SpAcDex particles that carry TPP. We studied their cellular uptake and light-dependent cytotoxicity in human KB cells, which are known to express high levels FRα [24,25].

## 2. Materials and Methods

KB cells (human cervix carcinoma cells derived from HeLa cells) were purchased from Cell Lines Service (Eppelheim, Germany). Cells were cultured in high-glucose Dulbecco′s modified Eagle′s medium (Gibco, Darmstadt, Germany) supplemented with 10% fetal calf serum (FCS), 1% penicillin/streptomycin, and 1% glutamine. FA (>99 %), tetraphenylporphyrin (TPP), and DAPI (4′,6-diamidino-2-phenylindole) nuclear staining reagent were obtained from Sigma–Aldrich (Steinheim, Germany). WST-1 reagent was purchased from Roche Applied Science (Mannheim, Germany). The mounting medium ProLong© Gold antifade reagent was obtained from life technologies (Darmstadt, Germany). Dextran (MW 9–11 kDa), folic acid, *N*-hydroxysuccinimide (NHS), *N*,*N*′-dicyclohexylcarbodiimide (DCC), and polyvinyl alcohol (MW 13–23 kDa, 87–89% partly hydrolyzed) as well as all salts and organic solvents were purchased from Sigma Aldrich. Fluorescamine was purchased from TCI (Zwijndrecht, Belgium).

### 2.1. Nanoparticle Preparation and Surface Modification

#### 2.1.1. Synthesis of Spermine-Modified Acetalated Dextran

The particle material, a spermine-modified and acetalated dextran (SpAcDex), was synthesized using a protocol previously described by Cohen et al. [5].

#### 2.1.2. Preparation of Empty and TPP-Loaded Particles

The preparation of empty and TPP-carrying (5,10,15,20-tetraphenyl-21H,23H-porphine) SpAcDex particles was adapted from a single emulsion evaporation method previously described by Bachelder et al. [3]. In short, 10 mg SpAcDex (MW 9–11 kDa) were dissolved in 400 mL ice-cold dichloromethane (DCM). Similarly, for the preparation of TPP-carrying particles, 1 mg was dissolved with 10 mg SpAcDex in 400 mL DCM (results in an initial feed of 100 µg TPP per mg SpAcDex). Afterwards, 2 mL polyvinyl alcohol (PVA) solution was added to the dissolved dextran solution, and an oil-in-water emulsion was prepared by sonicating the sample for 30 s. The prepared single emulsion was stirred overnight to evaporate the DCM, followed by the particle purification using ultracentrifugation (45,000 g, 20 min) and rinsing of the pellet with dd-H_2_O [double distilled water adjusted to pH 8 with triethylamine (TEA)]. Before lyophilization, 100 mL PVA (0.3% in dd-H_2_O pH 8) were added as cryoprotectant. The lyophilized particles were resuspended and characterized by size and zeta potential. The yield of the particles was ~65% (m/m) (referred to initial mass).

#### 2.1.3. Activation of Folic Acid for Nanoparticle Attachment

**Figure 1:**
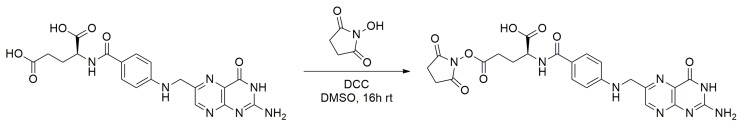
*N*,*N*′-Dicyclohexylcarbodiimide mediated activation of folic acid with *N*-hydroxy-succinimide.

Folic acid (0.4 g, 0.91 mmol) was dissolved in DMSO (6 mL) under argon atmosphere. *N*-Hydroxysuccinimide (0.22 g, 1.95 mmol) and *N*,*N*′-dicyclohexylcarbodiimide (0.39 g, 1.9 mmol) were added, and the reaction mixture was stirred at room temperature in the dark for 16 h (see Figure 1). The reaction solution was filtered, and the filtrate was poured in EtOAc (40 mL). The crude product was re-dissolved in DMSO and re-precipitated in EtOAc (5 x) to give FA-NHS (0.37 g, 75.3%) as a yellow solid. 1H-NMR (300 MHz, DMSO-d6, δ): 11.44 (s, 1H), 8.65 (s, 1H), 8.23 (d, J = 7.6 Hz, 1H), 7.71–7.59 (m, 2H), 6.94 (s, 3H), 6.70–6.60 (m, 2H), 4.49 (s, 2H), 4.44–4.28 (m, 1H), 2.80 (s, 4H), 2.37–2.23 (m, 1H), 2.21–1.83 (m, 2H). 13C-NMR (75 MHz, DMSO-d6, δ): 174.4, 174.2, 173.8, 173.2, 170.7, 169.0, 166.9, 156.7, 154.2, 151.3, 149.1, 129.5, 121.6, 111.7, 51.9, 46.4, 30.9, 25.9, 25.7; HRMS (ESI) m/z: [M + H] + calc. for C_23_H_22_N_8_O_8_, 539.47; found, 539.18.

#### 2.1.4. Nanoparticle Surface Modification with Folic Acid

**Figure 2:**
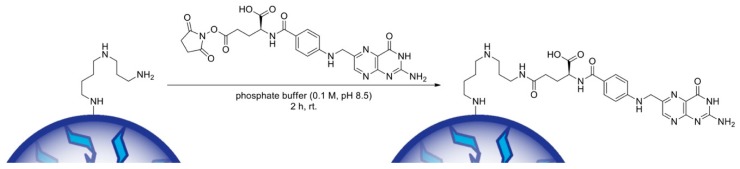
Surface modification of spermine functionality of spermine- and acetal-modified dextran nanoparticles (SpAcDex-NPs) (particles in blue) with *N*-hydroxysuccinimide-(NHS)-activated folic acid under buffered conditions.

Nanoparticles were suspended in phosphate buffer (0.1 m, pH 8.5), and a solution of FA-NHS was added to achieve a final particle concentration of 5 mg/mL and a final FA-NHS concentration of 4 mg/mL. The suspension was mixed for 2 h in the dark (see Figure 2). The modified particles were collected via centrifugation (30,000 g, 30 min, 20 °C) and washed three times with dd-H_2_O (pH 8). The particles were freeze dried for further storage. Before lyophilization, 10 µL PVA (0.3% in dd-H_2_O pH 8) per mg of the initial particle weight were added as cryoprotectant.

### 2.2. Nanoparticle Analysis

#### 2.2.1. Determination of Primary Surface Amines

The amount of primary amines on the particle surface was determined using a fluorescamine assay previously described by Foerster et al [12]. Briefly, empty SpAcDex particles were suspended in phosphate buffered saline (PBS) (4 mg/mL). Then, 125 µL PBS, 25 µL sample or hexylamine standard (19–40 μg/mL), and 50 µL fluorescamine dissolved in acetone (0.3 mg/mL) were mixed, and the emission of the fluorescamine was read using a Tecan Infinite® M200 Pro microplate reader (excitation λ = 380 nm, emission λ = 460 nm). Calculated with a calibration curve, 150 nmol amines per milligram particles were obtained.

#### 2.2.2. Quantification of Encapsulated TPP

TPP was quantified by a calibration curve. TPP was dissolved in DMF (dimethylformamide) in increasing concentrations, and its emission at 650 nm was measured after excitation with 420 nm. The particles were dissolved in DMF in different dissolving ratios to free TPP from the polymeric particles. The resulting TPP concentration was compared to the amount from the particle.

#### 2.2.3. Nanoparticle Size and Zeta Potential

Nanoparticle size was determined by dynamic light scattering (DLS) with a Malvern Zetasizer Nano ZS instrument. Evaluation of the data was performed with the Zetasizer software 6.20 and Mark-Houwink parameters. Empty and TPP-loaded SpAcDex particles were suspended in PBS (filtered 0.22 µm) at concentrations of approximately 25 µg/mL, and suspensions were sonicated as well as vortexed thoroughly for 20 s before each measurement. Each measurement consisted of 12 runs and was repeated three times.

The surface charge of the particles was measured by a Malvern Zetasizer Nano ZS instrument using a clear disposable zeta cell. Three measurements with 12 individual runs were performed at 25 °C. Particle samples were prepared at concentrations of 0.1 mg/mL in HEPES (4-(2-hydroxyethyl)-1-piperazineethanesulfonic acid) buffer (25 mM, pH 7.4). The refractive index (RI) of the dispersant (preset: water) was adjusted to 1.330 and the viscosity to 0.8872 cP with a dielectric constant of 78.5. The RI of the particle material dextran was set to 1.590. The data was analyzed by the model of Smoluchowski with the Malvern Zetasizer software 6.20 (Malvern, UK).

#### 2.2.4. Folic Acid Amount on Surface of Particles

After the chemical modification of the particle surface, a sample of the supernatant (20 µL) from the first centrifugation step was collected and diluted with phosphate buffer (pH 8.5, 480 µL). The absorption of the sample and a dilution of the initial FA sample was compared to a dilution of FA-NHS in phosphate buffer (pH 8.5). Absorption was measured in a 96-well plate (λ = 350 nm) as triplet (100 µL) using an Infinite® 200 PRO (Tecan, Männedorf, Switzerland) plate reader. The background of the buffer was subtracted from each measurement.

### 2.3. In Vitro Analysis of Nanoparticle Behavior

#### 2.3.1. Flow Cytometry

KB cells were plated in 12-well-plates (80,000 per well). After 24 h, cells were incubated for various times (1.5 h, 6 h, and 24 h) with 10 or 20 µg/mL of the different particles in medium in the absence and the presence of 1 mm FA. Afterwards, cells were washed with PBS, detached with trypsin, and fixed with ice cold paraformaldehyde (PFA, 4%). TPP fluorescence was measured of 30,000 cells in a BD© FACS calicur flow cytometer excited by 488 nm. Fluorescence was detected with a 650 nm LP filter (FL3-H). Images were processed with FlowJo_V10 (Ashland, OR, USA).

#### 2.3.2. Fluorescence Microscopy

KB cells were plated on cover slips (18 mm, Nr. 1.5) in 12-well-plates (80,000 per well). After 24 h, cells were incubated for various times (1.5, 6, and 24 h) with 20 µg/mL of the different particles in medium in the absence and the presence of an excess of 1 mm FA. Afterwards, cells were washed with PBS and fixed with ice cold paraformaldehyde (PFA, 4%). Fixed cells were incubated with DAPI solution (300 nm) in PBS for 5 min. Afterwards, they were washed several times with fresh PBS. The cover slips were put on mounting medium on object slides and analyzed with a TCS SP5 confocal fluorescence microscope using excitations at 405 nm for DAPI fluorescence and 560 nm for TPP. Images were taken and processed with Leica Application Suite X software and Image J©.

#### 2.3.3. Analysis of Cell Viability

KB cells were plated in 96-well plates (5000 per well). After 24 h, they were incubated for 1.5, 6, and 24 h with different amounts of dextran nanoparticles solved in medium in the absence and the presence of FA (1 mm). Afterwards, cells were washed with PBS and irradiated from the top of the cell culture dish without plastic cover for 15 min with an Osram halogen lamp (1000 W). The samples were placed at a distance of 33 cm corresponding to a dose rate of 375 W/m^2^ measured between 400 and 800 nm. After irradiation and washing with PBS, 100 µL of culture medium with 10 µL WST-1 reagent was added to every well. The tetrazolium dye in the WST-1 reagent was reduced to a formazan by mitochondrial enzymes, and the color changed from red to yellow in the presence of vital cells. The increase of the absorption at 450 nm measured directly after the addition of the reagent and exactly 2 h later was an indicator of the metabolic activity and therefore the viability of the cells. The relative viability was calculated as the ratio of the increase in absorption in treated cells and control cells (not exposed to any particles).

## 3. Results and Discussion

### 3.1. NP Preparation

Dextran based particles are a well-established drug delivery platform [3,4,5,6,8,11,12,26,27,28,29]. To compare the efficiency to deliver drugs for PDT both with and without targeting, we prepared different types of particles. Standard AcDex nanoparticles have no charged groups on the surface and therefore a low zeta potential. We prepared AcDex particles with an average diameter of 210 to 240 nm (Table 1). The spermine-modified AcDex variants (SpAcDex) are smaller than the AcDex particles with an average diameter of 130 to 180 nm. In addition, the zeta potential of the SpAcDex particles is increased compared to the AcDex because of the high number of exposed amines on the particle surface. The FA-modification of the SpAcDex particles leads to no significant change of the nanoparticle size, but the zeta potential is shifted towards a negative potential as a result of the lower amount of amines and the high number of carboxylic groups on the surface. The encapsulation of TPP results in an amount of 5.4 nmol (3.3 µg) per mg particle material in the case of AcDex particles and 7.6 nmol (4.7 µg) per mg particle material for SpAcDex nanoparticles. In both cases, the resulting loaded particles are smaller in size compared to the empty particles, which can be explained due to the additional hydrophobic interactions between TPP and the hydrophobic particle material. The amount of encapsulated TPP per particle material is comparable with previous loading efficiencies of hydrophobic payloads in dextran particles. Studies using polymeric and lipid-based materials report comparable loading efficiencies of 12 µg/mg [30,31] and 3.45 µg/mg [30,31], respectively.

### 3.2. Surface Modification of Nanoparticles with Folic Acid

Activation of FA for further coupling to the amino groups of spermine on the surface of SpAcDex and SpAcDex(TPP) nanoparticles was achieved by carbodiimide (DCC) mediated coupling of *N*-hydroxysuccinimide to the *γ*-terminal carboxylic terminus of the glutamic acid moiety. It is well described that this position is highly preferred over the alpha terminus, and no multiple attachment of NHS-groups can be observed [32]. The activated FA conjugate was then conjugated to the surface-amines of empty and TPP-loaded NPs under slightly basic buffer conditions. After attachment to the surface of the nanoparticles, the FA retains its receptor binding capacity and can be further used for receptor mediated endocytosis. The spermine chains on the particles serve as spacers between the dense particle surface and the FA molecules, providing additional flexibility of the targeting group.

The loss of positively charged amino groups on the particle surface can be qualitatively determined by the decrease of the observable surface charge. This allows a quantitative analysis of the attached amount of FA (Table 2). Similar to a method described elsewhere [33], the amount of remaining FA in the solution of the conjugation step was determined by the absorbance of the supernatant at *λ* = 350 nm. For this purpose, the difference of the absorbance of the supernatant before and after the reaction can be brought in relation to the amount of used particles. By this method, we determined an amount of FA on the particle surface of 196 nmol/mg for empty SpAcDex and 212 nmol/mg for drug-loaded SpAcDex(TPP) nanoparticles (see Table 2). These values are significantly higher than some known systems noted in literature, for example, as shown in studies by Das et al. (18 nmol/mg) and Fan et al. (33 nmol/mg) [34,35]. It can be concluded that the pre-activation of FA is an excellent step to enhance the coupling efficacy.

We conclude that, on average, every spermine spacer on the particle surface is carrying 1.33 FA molecules. This suggests that the primary amino groups of the spermine moiety reacted quantitatively, as did some of the secondary amino groups. The high degree of modification was further substantiated by the measurement of a relatively high negative zeta potential (see Table 1), which can be attributed to the lack of positively charged surface amines and the addition of carboxylic acids as part of the FA residues.

### 3.3. Cellular Uptake of Nanoparticles

The aim of this study was the targeted delivery of TPP with the help of folic acid modified particles. Therefore, we next compared the cellular uptake in KB cells of SpAcDex(TPP) and SpAcDex(TPP)-FA NPs in the presence and the absence of 1 mM FA, which serves as a competitor for receptor mediated uptake. Both FACS (flow cytometry) experiments and confocal fluorescence microscopy were performed to analyze the uptake into the cells. KB carcinoma cells were used, since they have been shown to strongly express FRα under our culture conditions [24,25].

#### 3.3.1. FACS-Analysis

**Figure 3:**
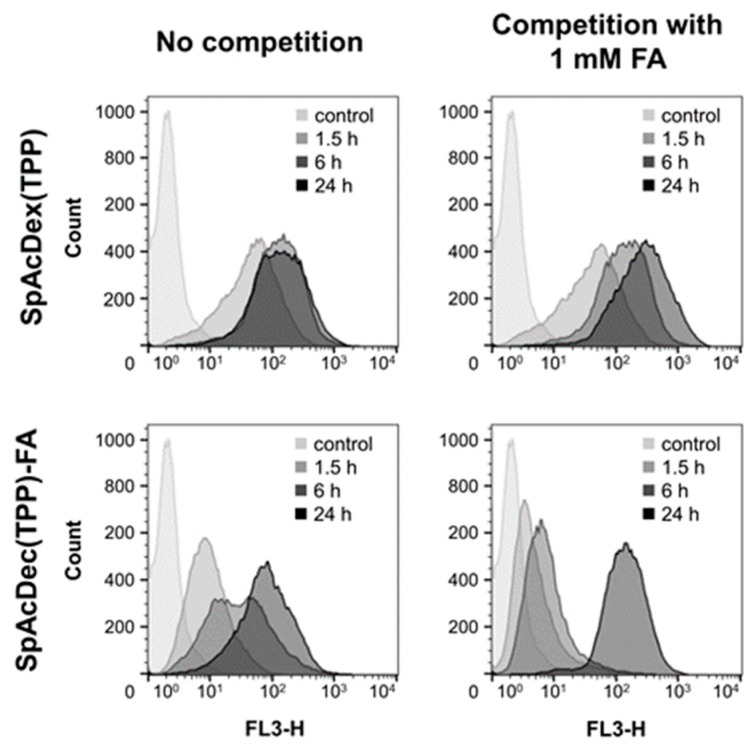
Time dependent uptake of 20 µg/mL TPP-loaded nanoparticles into KB cells in the absence or the presence of an excess of FA (1 mM) to compete for the FRα [fluorescence was detected with a 650 nm LP filter (FL3-H)]. SpAcDex particles without FA on the surface (top row) were taken up equally well in the absence or the presence 1 mM FA. However, the cellular uptake of SpAcDex particles with FA covalently attached on the surface (bottom row) is reduced in the presence of FA due to the competition for the FRα mediated endocytosis.

The FACS experiments show that unmodified SpAcDex(TPP) particles are rapidly taken up by KB cells. The finding is best explained by the high positive surface charge of the particles, which leads to a faster endocytosis than negative or neutral charged particles [11]. After a 6 h incubation time, all particles are taken up (Figure 3, top, left). Incubation with 1 mM FA does not change the uptake significantly, indicating that the uptake is not receptor-mediated (Figure 3, top, right). For the FA-modified particle SpAcDex(TPP)-FA, a slower, time-dependent cellular uptake was observed, which can be explained by the absence of the positive surface charge [36]. Importantly, when the same particle batch was incubated in a medium supplemented with 1 mM FA as a competitor for receptor-mediated uptake, a much lower intracellular fluorescence was observed at the early time points of 1.5 and 6 h (Figure 3, bottom, right). After 24 h, the fluorescence signal had the same intensity as in the cases without competition. These results indicate that the particles with FA on the surface are actively taken up by a FRα-mediated endocytosis, in contrast to the SpAcDex particles without FA on the surface, which are taken up non-specifically because of their high surface positive potential (see above).

#### 3.3.2. Confocal Fluorescence Microscopy

**Figure 4:**
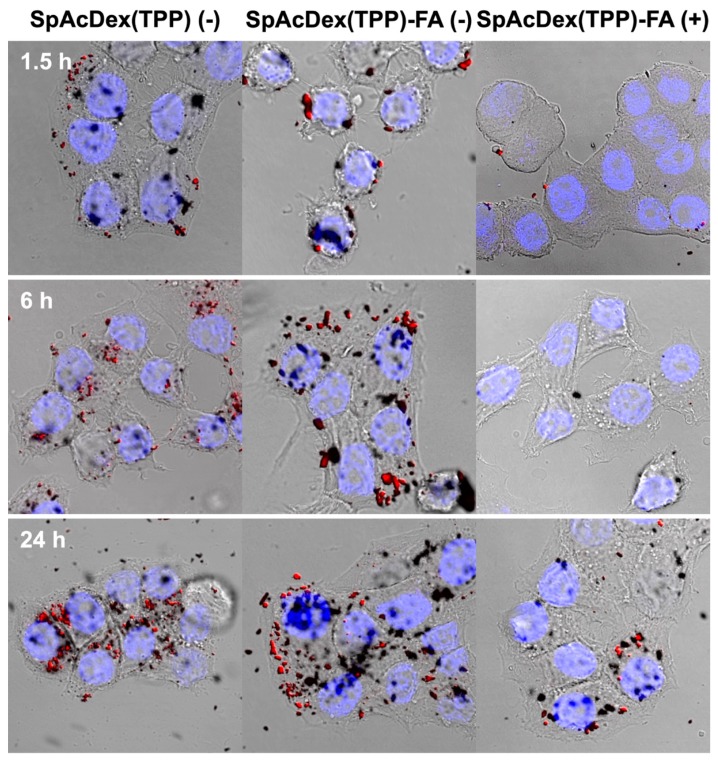
Confocal microscopy images of KB cells preincubated for 1.5, 6, and 24 h with SpAcDex(TPP) particles without and with FA conjugated on the surface. All particles were incubated in a concentration of 20 µg/mL. SpAcDex(TPP)-FA particles were additionally incubated without (-) and with (+) 1 mM folic acid as a competitor for FRα. Both ligand-free SpAcDex and FA-modified particles are time-dependently taken up by the cells (left and central columns). However, the uptake of SpAcDex(TPP)-FA particles in the presence of 1 mM FA is significantly reduced (right column). See Supplementary Data for single channel TPP, DAPI, and transmission images.

To conform the FACS results, we analyzed the cellular uptake in KB cells of SpAcDex(TPP) and SpAcDex(TPP)-FA particles in the presence of 1 mM free FA by confocal fluorescence microscopy (Figure 4).

For all particle types, the fluorescence signal was found to increase with time. The SpAcDex(TPP) particles with and without FA modification behave fairly similarly and show high update efficiency, which is in agreement with the results obtained by flow cytometry (Section 3.3.1). For the FA-modified particles, a slightly higher fluorescence signal can be observed at 6 and 24 h (Figure 4, central column) in comparison to the unmodified particles (Figure 4, left column). For the FA-modified particles, a clear influence of the FA competition (+) can be seen at all time points (Figure 4, right column). This confirms the conclusion from the flow cytometric analysis (Section 3.3.1) that the particles with FA on the surface are taken up selectively by FRα-mediated endocytosis. Both experiments indicate a selective targeting of KB-cells with FA-modified particles but not with unmodified particles.

### 3.4. Toxicity of TPP-Loaded Nanoparticles

The phototoxicities of SpAcDex(TPP) and SpAcDex(TPP)-FA NPs were analyzed by using a cell viability assay measuring the metabolic activity via intracellular formazan formation from a tetrazolium salt (WST-1). After incubation of the cells with the particles for various times, the cells were washed and subsequently irradiated with a halogen lamp emitting visible light (338 kJ/m^2^) for 15 min. We also analyzed the toxic effect of AcDex(TPP) particles. These particles have no positive charge and should be taken up by the cells with a much lower degree, and therefore should show no photoinduced cytotoxic effect at the investigated concentrations.

**Figure 5:**
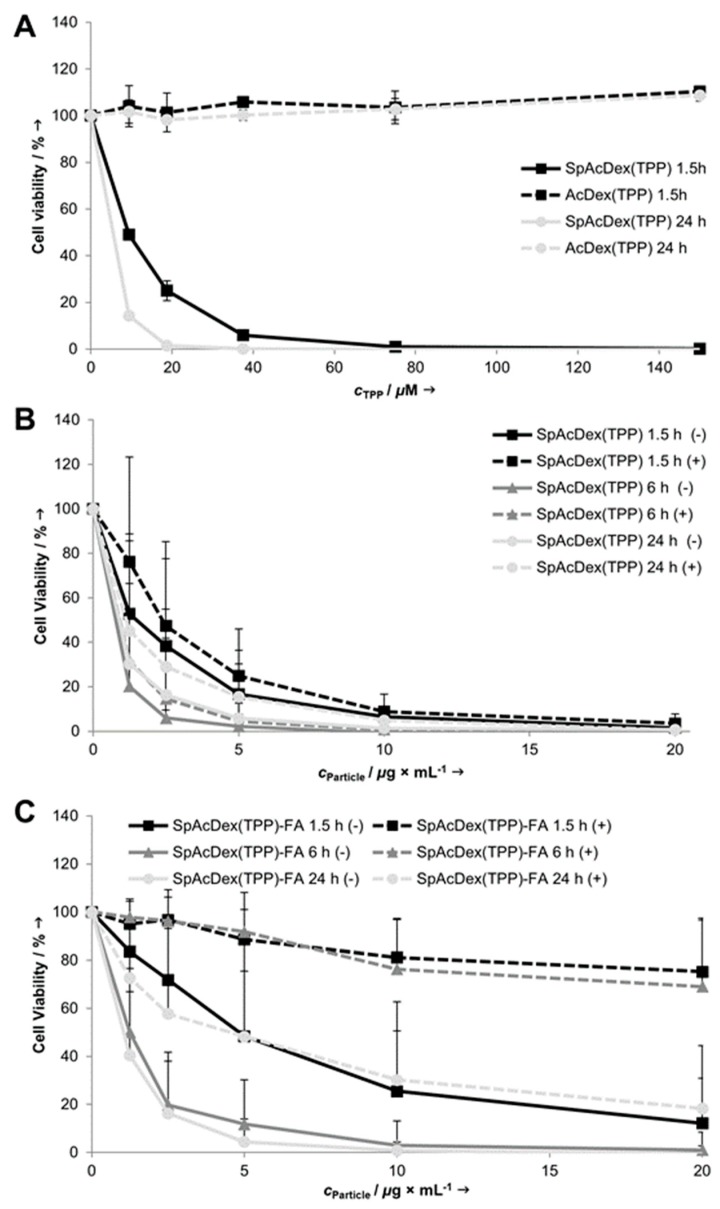
Cell viability of KB cells after preincubation with different TTP-loaded NPs for the indicated times and subsequent irradiation with visible light (338 kJ/m^2^). (**A**) AcDex(TPP) and SpAcDex(TPP) NPs. (**B**) SpAcDex(TPP) NPs with and without competition of 1 mM FA. (**C**) SpAcDex(TPP)-FA NPs with and without competition of 1 mM FA. Data in (**A**) are means of 2, and data in (**B**,**C**) are of 3-6 independent experiments + SD. AcDex(TPP) NPs show no phototoxic effects, whereas SpAcDex(TPP) NPs are highly phototoxic. SpAcDex(TPP)-FA NPs are somewhat less phototoxic, but their photoxicity—in contrast to that of SpAcDex(TPP) NPs—is strongly reduced when the preincubation was carried out in the presence of 1 mM FA.

The results of the WST-assay (Figure 5A) indicate that TPP-loaded SpAcDex particles have a concentration and an incubation time-dependent phototoxic effect on KB cells. In contrast, neutral AcDex(TPP) particles show no light-induced cytotoxic effect. This demonstrates that the cationic surface charge of SpAcDex particles facilitates a fast uptake by the KB cells, whereas the low surface charge of the AcDex(TPP) particles inhibits a cellular uptake. Figure 5B shows that the phototoxic effect of SpAcDex(TPP) particles is nearly independent of the presence (+) or the absence (-) of folic acid during preincubation. This indicates an unspecific (FRα-independent) uptake of SpAcDex(TPP) particles. In contrast, Figure 5C shows that the phototoxicity of folic acid-functionalized particles [SpAcDex(TPP)-FA] is strongly reduced when FA (1 mM) is present during the preincubation and competes with the particles for binding to the cellular FRα. The effect is particularly evident for the short incubation times (1.5 and 6 h). The cell viability stays even at high particle concentrations between 80–100%. Only after the longest preincubation time of 24 h phototoxicity becomes pronounced despite the presence of FA. These results demonstrate that the competition with free FA reduces the phototoxic effect of the SpAcDex(TPP)-FA particles strongly and thus indicates a specific FRα-mediated cellular uptake. Overall, the SpAcDex(TPP) particles show a remarkably high cytotoxicity at low particle and TPP concentrations compared to other recently published studies [31].

## 4. Conclusions

Our data indicates that it is possible to successfully encapsulate the photosensitizer TPP in biocompatible and biodegradable SpAcDex nanoparticles. The particle system stabilizes the hydrophobic drug and therefore minimizes the uncontrolled aggregation and precipitation under physiological conditions in therapeutic applications. Only under slightly acidic conditions, as can be found in endosomes and lysosomes, the acetals of the particle material are hydrolyzed, and the payload is released inside of the targeted cells. Therefore, no unspecific phototoxicity should be expected. 

The findings extend the areas of possible applications in photodynamic therapy beyond local irradiation. We were able to show that NPs with FA on the surface are taken up by cells through FRα-mediated endocytosis. This specific uptake can potentially be exploited for the selective elimination of cancer cells with FRα overexpression, reducing the overall side effects of radiation. This active targeting of our nanoparticles can further benefit from the overall higher passive accumulation in tumor tissue due to the EPR effect [22,23].

The high phototoxic potential needs to be verified for in vivo conditions. For these experiments, NPs with a PEG-layer and FA attached on the surface should be favorable because of the longer circulation time based on the PEG induced stealth effect. This should increase the contribution of the EPR effect and the active targeting, resulting in therapeutic NPs with low side effects in photodynamic chemotherapy.

## Figures and Tables

**Table 1:** Nanoparticle size (diameter) and surface charge of empty and 5, 10, 15, 20-tetraphenyl-21H, 23H-porphyrine-(TPP)-loaded particles determined by dynamic light scattering. Z-Average (Z-Ave) describes the mean size of all particles in the sample; the number represents the size of the particles forming the biggest population in the sample.

Nanoparticles	Numbe [nm]	Z-Ave [nm]	PDI	Zeta Potential [mV]
AcDex	181.3 ± 8.9	223.6 ± 1.1	0.084 ± 0.01	−1.47 ± 3.65
AcDex(TPP)	175.8 ± 3.5	211.8 ± 0.8	0.080 ± 0.01	−0.97 ± 3.80
SpAcDex	118.6 ± 7.2	172.7 ± 1.9	0.136 ± 0.02	19.2 ± 3.59
SpAcDex(TPP)	112.9 ± 6.3	158.8 ± 0.6	0.103 ± 0.01	11.0 ± 0.72
SpAcDex-FA	99.8 ± 9.7	146.9 ± 5.2	0.121 ± 0.02	−14.2 ± 6.80
SpAcDex(TPP)-FA	113.4 ± 5.3	137.0 ± 4.3	0.035 ± 0.03	−8.57 ± 0.20

**Table 2:** Determination of the amount of folic acid (FA) attached to the surface of empty and TPP-loaded NPs.

Nanoparticle	Surface Modification [nmol/mg]
SpAcDex-FA	196
SpAcDex-FA(TPP)	212

## Supplementary Materials

The following are available online at https://www.mdpi.com/2073-4360/11/5/896/s1, Figure S1: FA-NHS ^1^H-NMR, Figure S2: FA-NHS ^13^C-NMR, Figure S3: DLS curves for particles, Figure S4: Confocal images SpAcDex(TPP) without FA competition, Figure S5: Confocal images SpAcDex(TPP)-FA without FA competition, Figure S6: Confocal images SpAcDex(TPP)-FA with FA competition.
