# Supplementary material for: Receptor-mediated Uptake of Folic Acid-functionalized Dextran Nanoparticles for Applications in Photodynamic Therapy
Source: Polymers (Basel). 2019 May 16;11(5):896. doi: 10.3390/polym11050896 (PMC6572481; doi:10.3390/polym11050896)
Supplement: Supplementary file 1 [file polymers-11-00896-s001.pdf]

Receptor-mediated Uptake of Folic Acid-functionalized Dextran Nanoparticles for Applications in Photodynamic Therapy

Kathrin Butzbach[1], Matthias Konhäuser[1], Matthias Fach[2,], Denise N. Bamberger[1], Benjamin Breitenbach[1], Bernd Epe[1] and Peter R. Wich[1,3,4]*

[1] Institute of Pharmacy and Biochemistry, Johannes Gutenberg University, Staudingerweg 5, 55128 Mainz, Germany

[2] Department of Health Technology, Technical University of Denmark, Produktionstorvet Building 423, 2800 Lyngby, Denmark

[3] School of Chemical Engineering, University of New South Wales, Science and Engineering Building, Sydney, NSW 2052, Australia

[4] Australian Centre for NanoMedicine, University of New South Wales, Sydney, NSW 2052, Australia

phone: +61 2 9385 4308

email: p.wich@unsw.edu.au

web: www.wichlab.com

NMR Spectroscopy

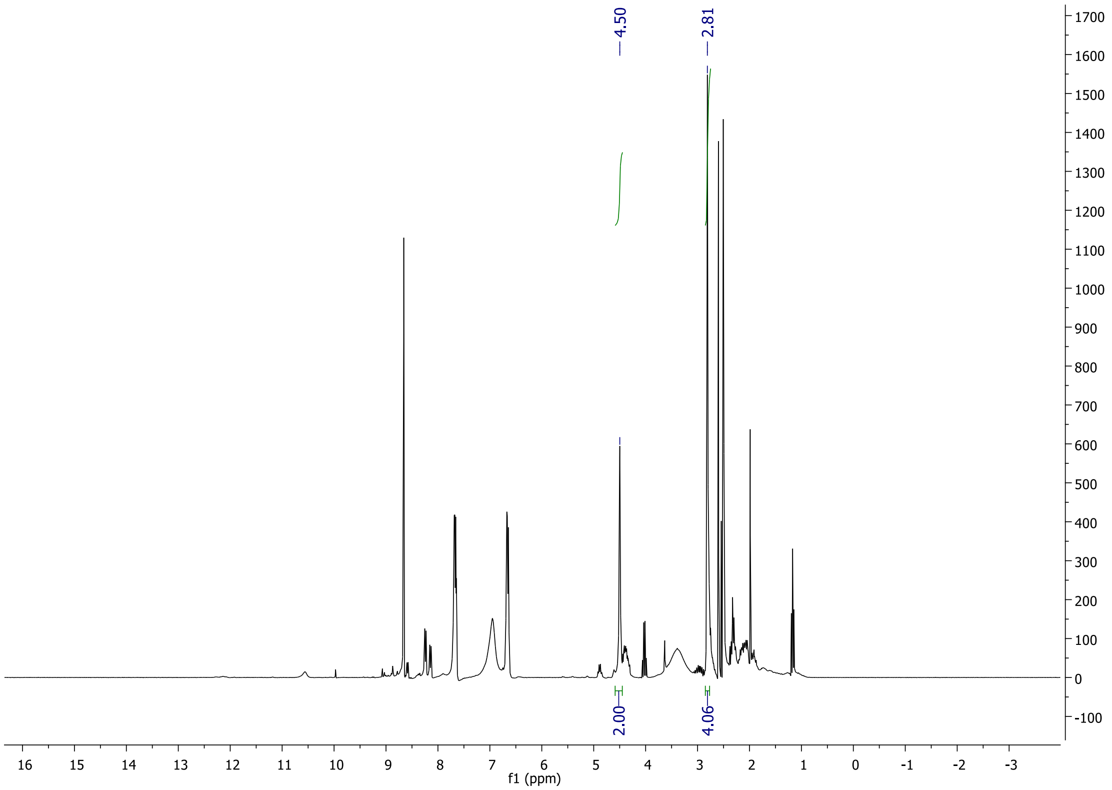

Figure S1: FA-NHS ^{1}H-NMR (300 MHz, DMSO-d_6)

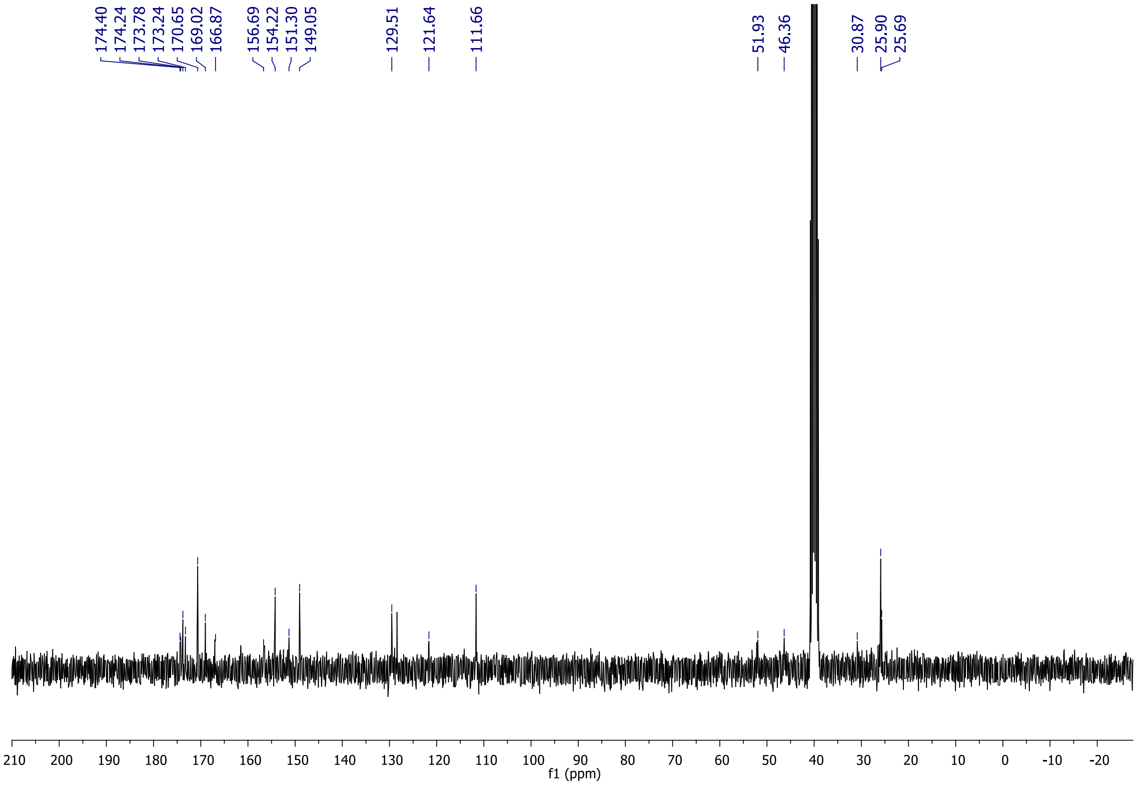

Figure S2: FA-NHS ^{13}C-NMR (75 MHz, DMSO-d_6)

Determination of Nanoparticle Size

Nanoparticle size was determined by dynamic light scattering (DLS) with a Malvern Zetasizer Nano ZS instrument. Evaluation of the data was performed with the Zetasizer software 6.20 and Mark-Houwink parameters. Empty and TPP-loaded Sp-Ac-DEX particles were suspended in PBS (filtered 0.22 µm) at concentrations of approx. 0.25 $\mu g \cdot mL^{-1}$ and suspensions were sonicated as well as vortexed thoroughly for 20 seconds before each measurement. Each measurement consisted of 12 runs and was repeated three times.

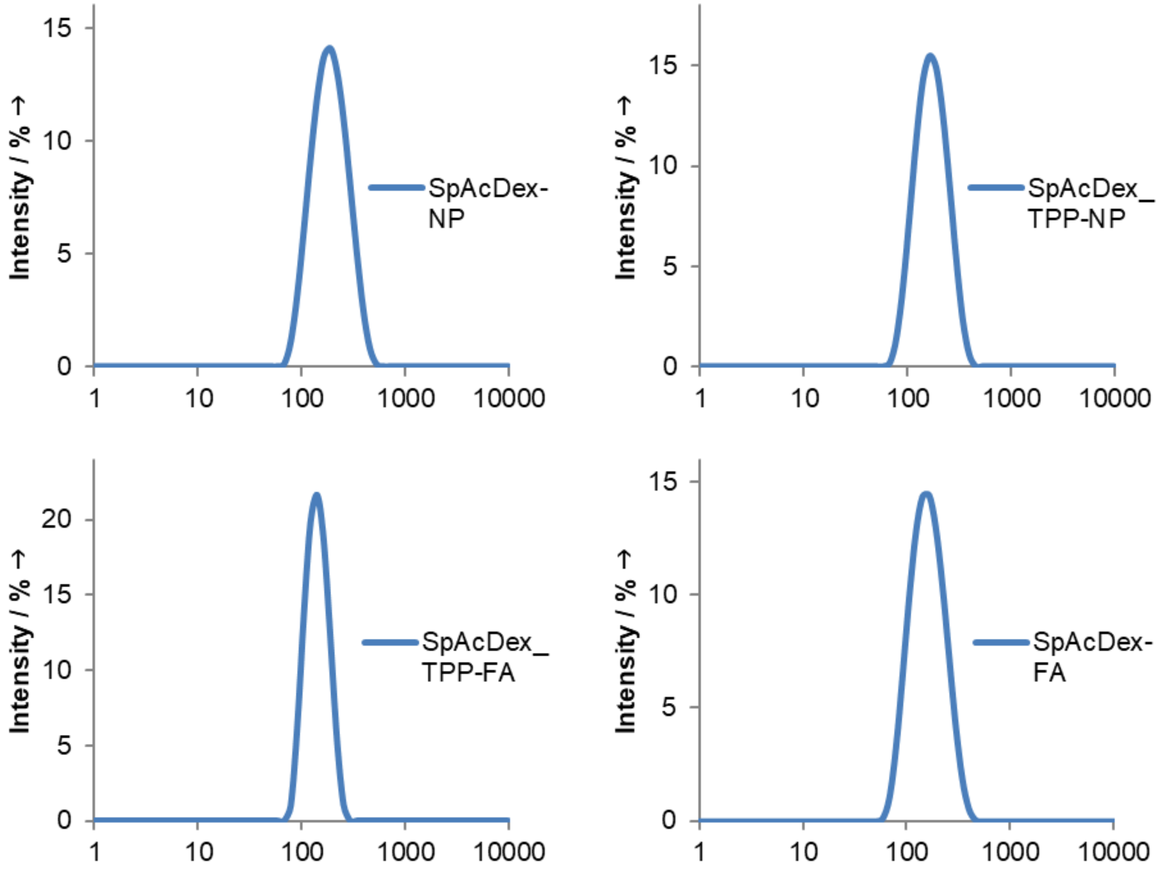

Figure S3: Nanoparticle size (diameter) of different SpAcDex particle types. Intensity describes the particle size distribution within the sample, depending on their scattering intensities.

Fluorescence Microscopy

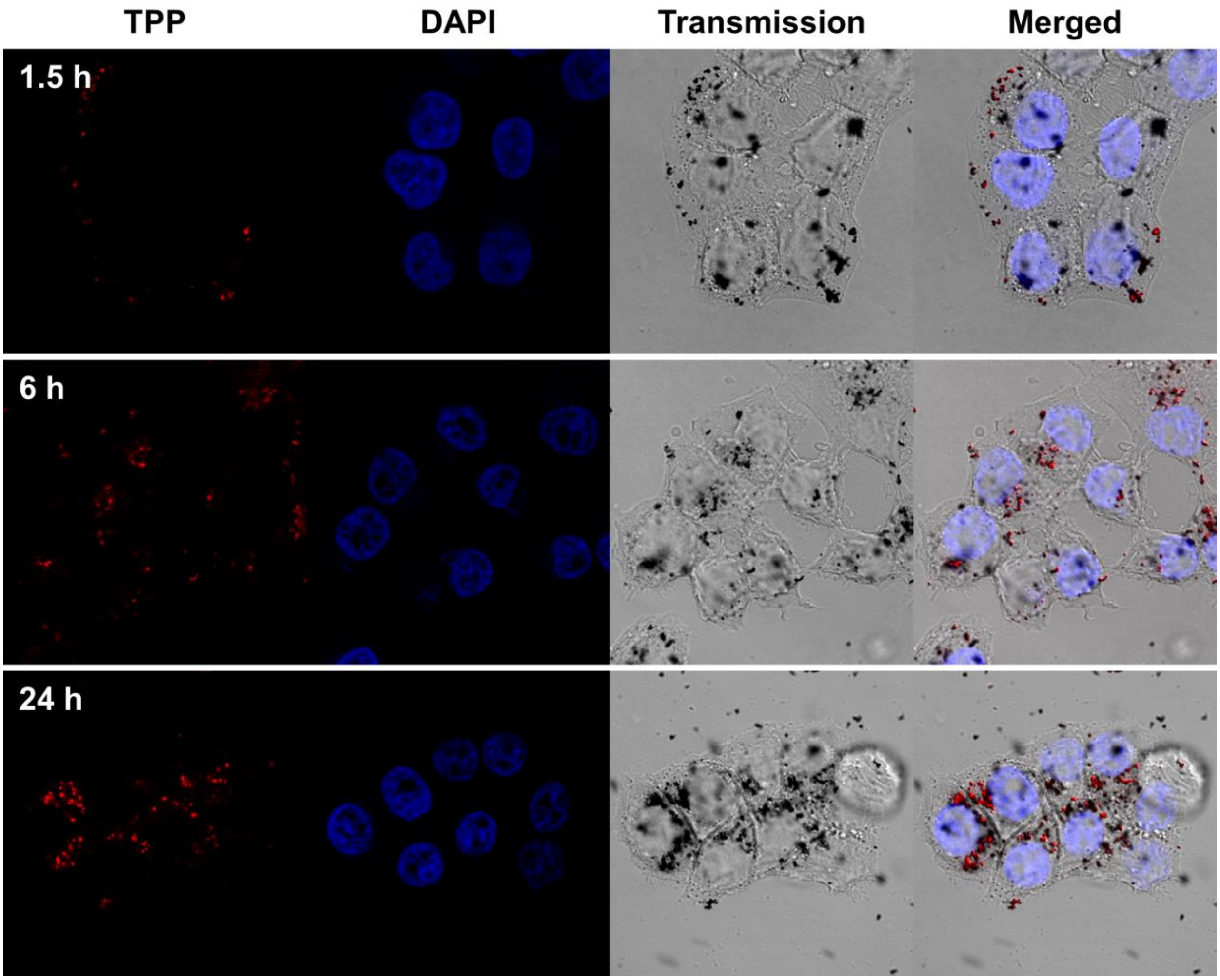

Figure S4: “Naked” SpAcDex(TPP) without FA competition

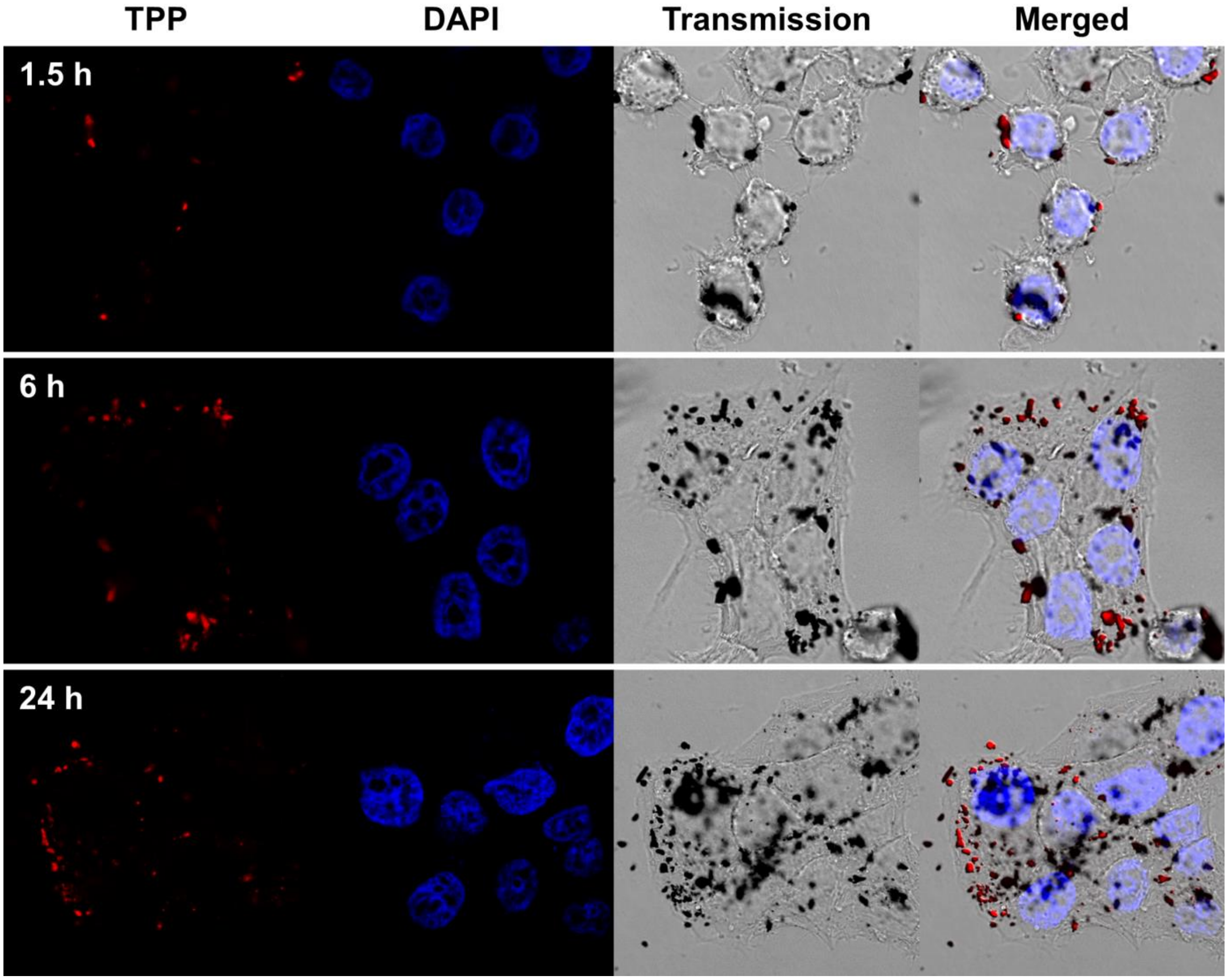

Figure S5: SpAcDex(TPP)-FA without FA competition

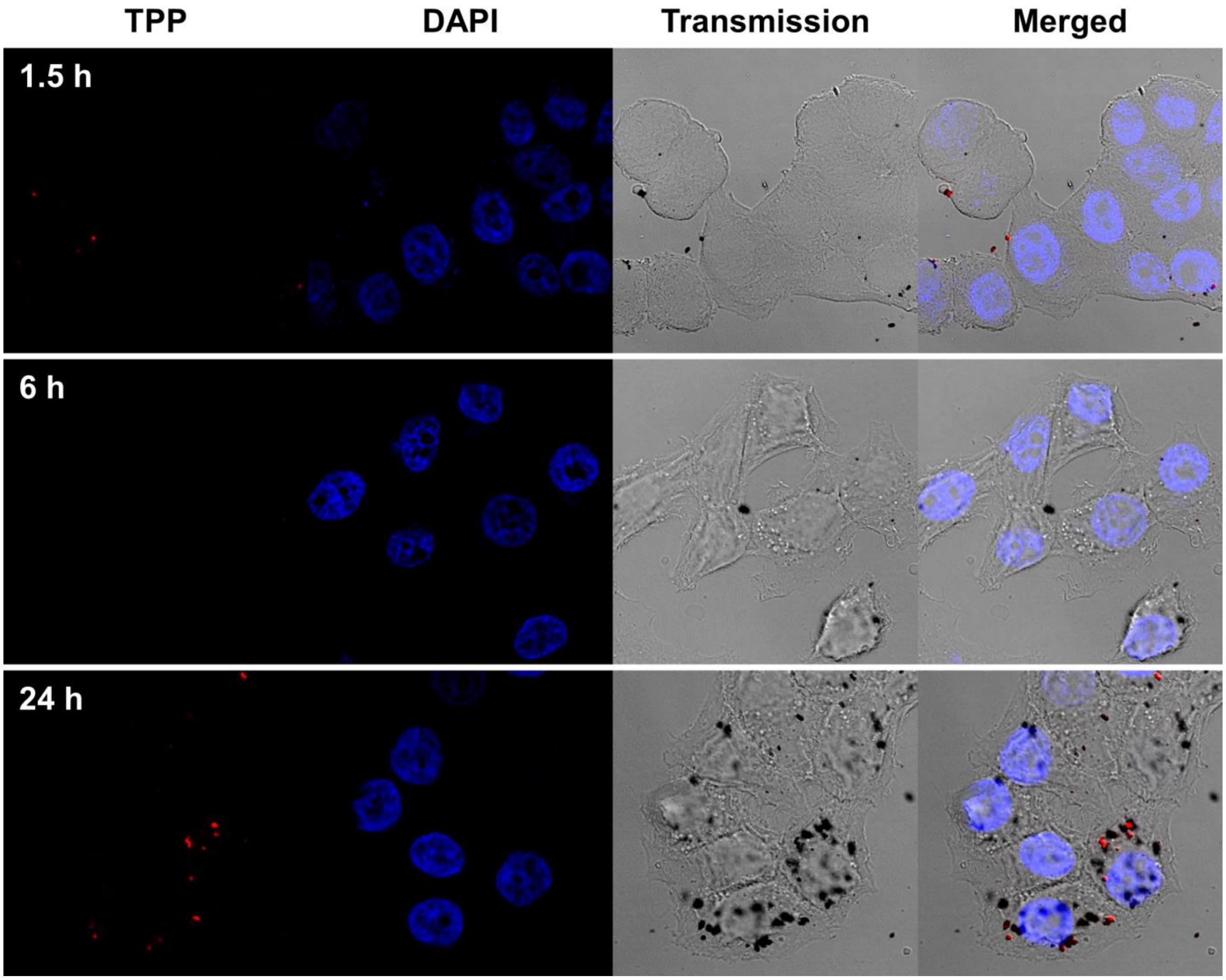

Figure S6: SpAcDex(TPP)-FA with FA competition
